# Cancer prognosis information-seeking among survivors and caregivers: findings from the National Cancer Institute’s Cancer Information Service

**DOI:** 10.1007/s00520-024-09089-8

**Published:** 2024-12-16

**Authors:** Ashley Wilson, Grace Huang, George Kueppers, Laura A. Dwyer, Paul K. J. Han, Robin C. Vanderpool

**Affiliations:** 1 Westat, Rockville, MD USA; 2 Behavioral Research Program, Division of Cancer Control and Population Sciences, National Cancer Institute, Rockville, MD USA; 3 Cape Fox Facilities Services, Chantilly, VA USA

**Keywords:** Prognosis, Health information-seeking, Cancer survivors, Caregivers

## Abstract

**Purpose:**

Receiving prognostic information is a well-documented need for cancer survivors and caregivers. However, little is known about these two groups’ prognosis information-seeking outside of discussions with healthcare providers. This study examined survivors’ and caregivers’ prognosis-related inquiries using data from the National Cancer Institute’s Cancer Information Service (CIS).

**Methods:**

Using an analytic sample of 81,154 survivors and caregivers, descriptive statistics explored differences between prognosis and non-prognosis inquiries made by each group over a 6-year period (September 2018–August 2024). Logistic regressions identified factors that were associated with odds of a prognosis inquiry among both cancer survivors and caregivers.

**Results:**

A higher proportion of caregivers (62%) made prognosis inquiries to the CIS compared to survivors (38%). Among both groups, telephone and instant chat were commonly used to contact the CIS with prognosis questions. Prognosis inquiries were more likely among survivors and caregivers who contacted the CIS in Spanish and whose inquiries centered on staging, post-treatment, or end-of-life phases of the cancer continuum. For both groups, prognosis inquiries were more likely to occur in the context of discussions about chemotherapy and general questions about cancer treatment. Discussion of prognosis as related to specific cancer sites was variable across survivors and caregivers.

**Conclusion:**

Findings may inform the development and targeting of messages to support cancer prognosis information-seeking among survivors and caregivers. Disseminating accessible, language-concordant prognostic information that accounts for survivors’ and caregivers’ respective information-seeking needs is merited. Efforts may contribute to enhancing prognostic understanding, supporting discussions with providers, and improving psychosocial outcomes.

## Introduction

Patient-centered communication and information provision are widely recognized as important parts of clinical and supportive care for cancer survivors and caregivers. Among individuals diagnosed with cancer, studies have shown that higher levels of met information needs are associated with positive psychosocial outcomes such as improved health-related quality of life and lower levels of anxiety and depression [1–6] as well as greater patient engagement, informed decision-making, and adherence to treatment [3, 7, 8]. However, those with cancer—regardless of their time since diagnosis or current disease status—must navigate and manage uncertainties related to their prognosis.

As defined by the National Cancer Institute (NCI), cancer prognosis refers to “the likely outcome or course of a disease; the chance of recovery or recurrence” [9]. Not unexpectedly, information about prognosis is a well-documented need of individuals affected by cancer. Cancer patients’ information needs may encompass various prognostic estimates, including the likelihood of cure, survival, recurrence of cancer, and other future health conditions [10]. Although some individuals prefer not knowing their prognosis [11], most value prognostic information during all phases of the cancer experience [12–14], and others report wanting more prognostic information than they received [15]. Survivors’ prognosis information needs are also situated within a broader set of supportive care needs, including managing uncertainties about their disease trajectory and navigating emotional experiences, which may include feelings of depression or anxiety [8, 16].

Caregivers also report unmet cancer prognostic information needs [17, 18]. Although caregivers are often present at medical appointments and participate in discussions and decision-making with both their loved ones and healthcare providers, caregivers’ information needs and coping strategies can differ from those of cancer survivors [19]. Caregivers’ information needs may be informed by their independent thoughts, beliefs, and ideas about the prognosis of their loved ones and appropriate actions in response to prognostic information [20]. Survivors and caregivers generally both prefer high levels of information, but as the illness progresses, caregivers may want even more prognostic information to better support the survivor [18, 21]. Specifically, prognostic information may enable caregivers to obtain more timely care for their loved ones (e.g., palliative care, hospice), while also enabling survivors to take appropriate actions to manage their illness [20]. In addition to their personal cancer information needs, caregivers may seek information on behalf of patients to provide informational support and aid in their decision-making [16, 22].

Among healthcare providers, approaches to effectively communicate prognosis to survivors and their caregivers vary greatly. Clinical communication in cancer care settings has several functions, including exchanging information [8]. Yet, exchanging information about prognosis is challenging for several reasons. Discussing prognosis is a complicated task that can be emotionally taxing for oncologists [23]. Moreover, these discussions are also difficult given the inherent uncertainties of applying prognostic estimates to individuals, variability in survivors’ preferences for—and volume of—prognostic information, and the task of communicating prognosis information with both accuracy and sensitivity in a way that balances patients’ need for knowledge with their need for hope [8, 24]. These communication challenges may drive survivors and caregivers to pursue prognostic information from additional sources beyond physicians and other clinical providers. For example, studies show that survivors and caregivers are increasingly seeking cancer information from social networks, healthcare organizations, public health agencies, and the internet [21, 25–28]. In addition, survivors and caregivers may contact national cancer information programs, such as NCI’s Cancer Information Service (CIS), a federally funded, United States-based program established in 1975 to help disseminate trusted cancer information to health professionals, patients, caregivers, and the public in English and Spanish via multiple communication channels [29]. Studies have found that people affected by cancer also seek a wide range of information from cancer information services in Australia and Germany [30, 31].

Considering the complex information environment survivors and caregivers must navigate to understand their prognosis and make clinical and other major life decisions, additional research is needed to examine their prognostic-related inquiries. Fortunately, CIS data collection procedures allow for real-time documentation and analysis of cancer information needs of those contacting the free, confidential service. Modeled after prior CIS descriptive assessments of specific cancer information-seeking needs (e.g., childhood cancer, cancer-related COVID-19 concerns, cannabis, clinical trials) [32–35], the current study explored prognostic information-seeking from cancer survivors and caregivers over a 6-year period. Specifically, the purpose of this analysis was to understand these two groups’ prognosis inquiries as compared to non-prognosis inquiries as well as examine factors that predict prognosis inquiries among cancer survivors and caregivers. The overarching goal was to provide insights that may inform the development and delivery of health information that is responsive to the prognostic information needs of these two groups of individuals affected by cancer.

## Methods

Using an electronic contact record form, trained information specialists code each CIS interaction to document characteristics of the inquiry including the type of individual contacting the CIS, the phase of the cancer continuum the inquiry is related to, and cancer-related topics, such as prognosis, which were discussed during the inquiry. The CIS defines prognosis inquiries as questions about the prognosis for cancer at any stage including the likelihood of recurrence as well as survival statistics.

### Sample

**Fig. 1:**
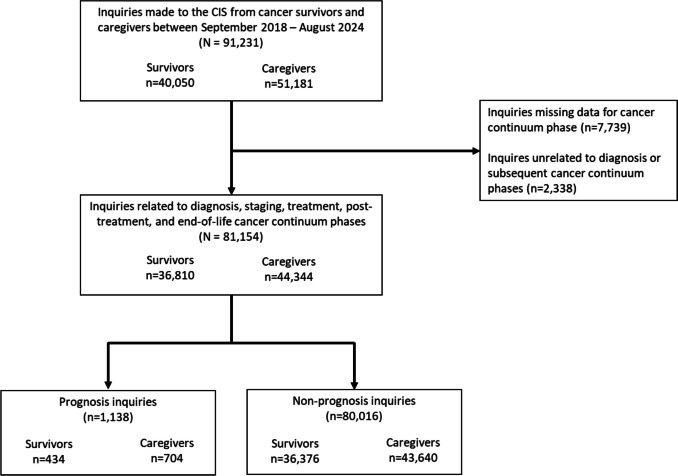
Flow chart highlighting inclusion and exclusion criteria as applied to the CIS analytic sample

Between September 2018 and August 2024, the CIS received 91,231 inquiries from cancer survivors and caregivers. Caregivers include CIS users who self-identify as a spouse, friend, or relative of a diagnosed cancer survivor. Our analysis adopts the NCI Office of Cancer Survivorship’s definition of a cancer survivor, which states that “an individual is considered a cancer survivor from the time of diagnosis, through the balance of his or her life” [36]. Among inquiries from either a survivor or caregiver, a subset of inquiries focused on the diagnosis and subsequent phases of the cancer care continuum was analyzed. This resulted in an analytic sample of 81,154 CIS inquiries received from cancer survivors and caregivers, with 1138 of these inquiries including an inquiry about prognosis (Fig. 1).

### Measures

Several categorical variables were used to identify characteristics of prognostic information-seeking including *language of service* (English, Spanish), *communication channel* used to contact the CIS (email, LiveHelp [instant messaging], social media, telephone), and other topics referenced during the CIS inquiry (phase of the cancer continuum, cancer site(s), other concurrent topics of discussion, and referrals given by CIS staff). The *phase of the cancer continuum* was coded into five categories (diagnosis, staging, treatment, post-treatment, end-of-life). Related to *cancer site(s)*, the following sites were the subject of at least 30 prognosis inquiries: breast, digestive/gastrointestinal, genitourinary, gynecologic, head and neck, hematologic, lung, musculoskeletal, or neurologic cancers.

We also examined the five most frequent *concurrent topics* that were the subject of the CIS interaction alongside prognosis, as coded by CIS information specialists. These included (1) chemotherapy; (2) coping (emotional coping strategies, emotional support); (3) finding any type of healthcare service, including support, transportation, or lodging services; (4) general cancer questions (topics related to cancer in general that are not specific to a type of treatment); and (5) general treatment questions (questions about treatment in general that are not specific to a type of treatment). In response to prognosis-related inquiries, CIS information specialists commonly give *referrals* to healthcare professionals (doctors, nurses, pharmacists, social workers, or other health practitioners), healthcare facilities, NCI-designated cancer centers, and other support organizations (e.g., community services, organizations, state, local, and federal government programs other than NCI), and we also analyzed associated referral data.

### Data analysis

Descriptive statistics were run separately for cancer survivors and caregivers to describe the total sample alongside characteristics of prognosis and non-prognosis inquiries. To understand factors associated with prognostic inquiries, we used logistic regression analyses on the full sample of survivors (*n* = 36,810) and caregivers (*n* = 44,344), respectively. Language of service, CIS communication channel, content of the discussion (phase of the cancer continuum, cancer site(s), and other concurrent discussion topics) were included as independent variables. All analyses were conducted in STATA/SE 15.1 (StataCorp LLC, College Station, TX). As CIS programmatic data are fully deidentified, this study was deemed non-human subjects research by Westat (No. 00005551) and National Institutes of Health (No. 568158) Institutional Review Boards.

## Results

Overall, prognosis discussions were a small proportion of post-diagnosis inquiries made to the CIS from survivors (1.2%) and caregivers (1.6%) over the 6-year study period. Among the 1138 total prognostic inquiries made by survivors and caregivers, 38.1% originated from cancer survivors and 61.9% from caregivers.

### Survivors’ prognosis inquiries

Descriptive statistics for cancer survivors are shown in Table 1. The 434 prognosis inquiries received from cancer survivors were predominantly English language (86.9%) and received via telephone (56.0%) or LiveHelp (36.2%). Compared to non-prognosis inquiries, prognosis inquiries from survivors were more likely to be made in Spanish (13.1% vs. 8.5%). A higher proportion of survivor prognosis inquiries (as compared to non-prognosis inquiries) came from LiveHelp (36.2% vs. 28.4%) and a lower proportion came from email (6.0% vs. 10.4%).

**Table 1:** Characteristics of prognosis and non-prognosis inquiries made to the CIS by cancer survivors (September 2018–August 2024)

	Prognosis	Non-prognosis	Total	*χ*^2^, *p*-value
*N* (%)	434 (1.2)	36,376 (98.8)		
	**%**	**%**	**%**	
Language				*χ*^2^ = 11.91,* p* = 0.00*
English	86.87	91.52	91.47	
Spanish	13.13	8.48	8.53	
Communication channel				*χ*^2^ = 19.06, *p* = 0.00*
Email	5.99	10.39	10.34	
LiveHelp	36.18	28.36	28.45	
Social media	1.84	1.36	1.37	
Telephone	55.99	59.88	59.84	
Cancer continuum phase				*χ*^2^ = 57.46, *p* = 0.00*
Diagnosis	4.84	5.09	5.09	
Staging	9.45	3.62	3.69	
Treatment	64.75	74.30	74.19	
Post-treatment	19.12	16.38	16.41	
End-of-life	1.84	0.60	0.62	
Cancer sites^1^				
Breast	19.59	22.17	22.14	*χ*^2^ = 1.66, *p* = 0.20
Digestive/gastrointestinal	12.21	14.38	14.36	*χ*^2^ = 1.64 *p* = 0.20
Genitourinary	18.66	17.08	17.10	*χ*^2^ = 0.76, *p* = 0.39
Gynecologic	11.75	7.93	7.98	*χ*^2^ = 8.53, *p* = 0.00*
Head and neck	3.92	3.18	3.19	*χ*^2^ = 0.75, *p* = 0.39
Hematologic	12.90	9.59	9.63	*χ*^2^ = 5.43, *p* = 0.02*
Lung	7.37	6.16	6.18	*χ*^2^ = 1.08, *p* = 0.30
Musculoskeletal	1.61	2.05	2.05	*χ*^2^ = 0.42, *p* = 0.52
Neurologic	2.30	1.69	1.69	χ^2^ = 0.99, *p* = 0.32
Discussion topics^1^				
Chemotherapy	6.91	2.77	2.82	*χ*^2^ = 26.90, *p* = 0.00*
Coping	5.76	3.61	3.64	*χ*^2^ = 5.65, *p* = 0.02*
Finding healthcare services	6.22	19.29	19.14	*χ*^2^ = 47.34 *p* = 0.00*
General cancer questions	15.90	13.50	13.52	*χ*^2^ = 2.12, *p* = 0.15
General treatment questions	22.35	14.26	14.35	*χ*^2^ = 22.84, *p* = 0.00*

*Statistically significant values (*α* = 0.05)

^1^Cancer types and discussion topics referenced during CIS inquiries are not mutually exclusive and therefore do not add up to 100%

Among survivors, there were noted differences in the proportions of prognosis inquiries compared to non-prognosis inquiries when referencing staging (9.5% vs. 3.6%), post-treatment (19.1% vs. 16.4%), and end-of-life (1.8% vs. 0.6%). Common cancer sites that survivors referenced during prognosis inquiries were breast cancer (19.6%), genitourinary cancer (18.7%), hematologic cancer (12.9%), digestive/gastrointestinal cancer (12.2%), and gynecologic cancer (11.8%). Compared to survivors’ non-prognosis inquiries, survivors’ prognosis discussions were significantly more likely to reference gynecologic cancer and hematologic cancer. Survivors’ prognosis inquiries were also more likely to focus on concurrent topics related to chemotherapy, general cancer treatment, and coping as compared to non-prognosis inquiries, and less likely to focus on finding healthcare services. Most survivors who made prognosis inquiries were referred by CIS information specialists to healthcare professionals (87.6%), followed by national or community organizations (21.4%) (data not shown).

### Predicting prognosis inquiries among survivors

As shown in Table 2, results from the logistic regression analysis indicate that among cancer survivors, the odds of a prognostic inquiry were greater for CIS inquiries conducted in Spanish versus English (OR = 1.69, 95% CI [1.26, 2.28]). Compared to the telephone service, the odds of a prognosis inquiry were greater with the use of LiveHelp (OR = 1.26, 95% CI [1.02, 1.56]) and lower with the use of email (OR = 0.56, 95% CI [0.37, 0.85]). Aspects of concurrent discussion content (cancer continuum phase, cancer sites, other topics) were also associated with the odds of a prognosis inquiry. For example, as shown in Table 2, survivors’ odds of a prognosis inquiry were over four times higher if their inquiry discussed end-of-life as compared to treatment (OR = 4.10, 95% CI [1.98, 8.50]). Discussions specifying genitourinary, gynecologic, hematologic, and lung cancers also had higher odds of including an inquiry about prognosis (ORs ≥ 1.45). Survivors’ inquiries that included discussion of chemotherapy were associated with greater odds of a prognostic inquiry as compared to inquiries that did not discuss chemotherapy (OR = 2.70, 95% CI [1.84, 3.97]). The odds of a prognosis inquiry were also higher with the presence of general treatment questions (OR = 1.70, 95% CI [1.34, 2.15]). In contrast, the odds of a survivor prognosis inquiry were lower when the inquiry also focused on finding healthcare services, as compared to those that did not discuss healthcare services (OR = 0.28, 95% CI [0.19, 0.42]).

**Table 2:** Logistic regressions of factors associated with prognosis inquiries to the CIS among cancer survivors (*n* = 36,810)

	OR	95% CI	
Language			
Spanish	1.69*	1.26	2.28
English (ref.)	-	-	-
Communication channel			
LiveHelp	1.26*	1.02	1.56
Email	0.56*	0.37	0.85
Social media	1.19	0.57	2.48
Telephone (ref.)	-	-	-
Cancer continuum phase			
Diagnosis	1.27	0.81	1.99
Staging	3.13*	2.22	4.41
Post-treatment	1.50*	1.16	1.93
End-of-life	4.10*	1.98	8.50
Treatment (ref.)	-	-	-
Cancer sites^1^			
Breast	1.15	0.84	1.58
Digestive/gastrointestinal	1.16	0.82	1.66
Genitourinary	1.45*	1.05	2.00
Gynecologic	1.95*	1.36	2.79
Head and neck	1.60	0.94	2.74
Hematologic	1.88*	1.32	2.67
Lung	1.62*	1.07	2.46
Musculoskeletal	1.09	0.50	2.36
Neurologic	1.94	1.00	3.78
Discussion topics^1^			
Chemotherapy	2.70*	1.84	3.97
Coping	1.50	1.84	3.97
Finding healthcare services	0.28*	0.19	0.42
General cancer questions	0.99	0.76	1.29
General treatment questions	1.70*	1.34	2.15

*Statistically significant values (*α* ≤ 0.05)

^1^For each cancer site and discussion topic, the reference category is the absence of that specific cancer site or discussion topic within a CIS inquiry. For example, the reference category for breast cancer is a CIS inquiry that did not mention breast cancer

### Caregivers’ prognosis inquiries

Descriptive statistics for caregivers are shown in Table 3. Caregivers’ prognosis inquiries were predominantly received in English (76.3%), although the proportion of Spanish language contacts was nearly double for inquiries about prognosis than for non-prognosis inquiries (23.7% vs. 12.5%). Half of caregivers’ prognosis inquiries were received via LiveHelp (49.6%), followed by telephone (39.2%). Among caregivers, the use of communication channels differed for prognosis versus non-prognosis inquiries, with prognosis inquiries being communicated more commonly via LiveHelp (49.6% vs. 35.6%) and less commonly via telephone (39.2% vs. 48.9%) and email (9.7% vs. 13.7%). Caregivers’ prognosis and non-prognosis inquiries most often referenced cancer treatment (70.3% and 81.5%, respectively). However, when comparing caregivers’ prognosis versus non-prognosis inquiries, diagnosis, staging, post-treatment, and end-of-life were more common in prognosis discussions. The three most common cancer sites that caregivers referenced in their prognosis inquiries were digestive/gastrointestinal (25.6%), genitourinary (13.2%), and hematologic (11.5%). Compared to non-prognosis inquiries, caregivers’ prognosis inquiries were more likely to concern genitourinary cancer (*χ*^2^ = 8.84, *p* = 0.00) and less likely to be about breast cancer (*χ*^2^ = 6.42, *p* = 0.01). Caregivers were also significantly more likely to reference chemotherapy, other treatment questions, and general cancer questions during prognosis inquiries as compared to non-prognosis inquiries (*χ*^2^ ≥ 12.61, ps < 0.001) and less likely to reference finding healthcare services (*χ*^2^ = 97.56, *p* < 0.001). Most caregivers who made prognosis inquiries were referred by CIS information specialists to health professionals (86.2%) or national/community organizations (16.2%) (data not shown).

**Table 3:** Characteristics of prognosis and non-prognosis inquiries made to the CIS by caregivers (September 2018–August 2024)

	Prognosis	Non-prognosis	Total	*χ*^2^, *p*-value
*N* (%)	704 (1.6)	43,640 (98.4)	44,344	
	%	%	%	
Language				*χ*^2^ = 78.01,* p* = 0.00*
English	76.28	87.46	87.28	
Spanish	23.72	12.54	12.72	
Communication channel				*χ*^2^ = 56.70, *p* = 0.00*
Email	9.66	13.71	13.65	
LiveHelp	49.57	35.60	35.82	
Social media	1.56	1.79	1.79	
Telephone	39.20	48.90	48.75	
Cancer continuum phase				*χ*^2^ = 106.16, *p* = 0.00*
Diagnosis	7.11	6.35	6.36	
Staging	8.24	2.77	2.86	
Treatment	70.31	81.51	81.33	
Post-treatment	5.82	4.61	4.63	
End-of-life	8.52	4.76	4.82	
Cancer sites^1^				
Breast	8.10	11.12	11.07	*χ*^2^ = 6.42, *p* = 0.01*
Digestive/gastrointestinal	25.57	23.08	23.12	*χ*^2^ = 2.40 *p* = 0.12
Genitourinary	13.21	9.84	9.89	*χ*^2^ = 8.84, *p* = 0.00*
Gynecologic	7.39	6.98	6.99	*χ*^2^ = 0.18, *p* = 0.68
Head and neck	4.26	3.52	3.54	*χ*^2^ = 1.10, *p* = 0.29
Hematologic	11.51	9.73	9.76	*χ*^2^ = 2.47, *p* = 0.12
Lung	9.94	9.25	9.26	*χ*^2^ = 0.40, *p* = 0.53
Musculoskeletal	4.55	3.70	3.71	*χ*^2^ = 1.38, *p* = 0.24
Neurologic	4.83	6.28	6.26	*χ*^2^ = 2.49, *p* = 0.12
Discussion topics^1^				
Chemotherapy	4.55	2.45	2.48	*χ*^2^ = 12.61, *p* = 0.00*
Coping	5.26	3.86	3.89	*χ*^2^ = 3.60, *p* = 0.06
Finding healthcare services	9.23	25.54	25.28	*χ*^2^ = 97.56 *p* = 0.00*
General cancer questions	21.16	13.34	13.46	*χ*^2^ = 36.45, *p* = 0.00*
General treatment questions	28.13	16.49	16.67	*χ*^2^ = 67.54, *p* = 0.00*

*Statistically significant values (*α* = 0.05)

^1^Cancer types and discussion topics referenced during CIS inquiries are not mutually exclusive and therefore do not add up to 100%

### Predicting prognosis inquiries among caregivers

As presented in Table 4, logistic regression results for caregivers show that the odds of a prognosis inquiry were higher for inquiries made in Spanish (OR = 2.05, 95% CI [1.69, 2.47]) and received through LiveHelp (OR = 1.44, 95% CI [1.22, 1.71]) as compared to telephone. Compared to inquiries referencing the phase of cancer treatment, caregivers were more likely to inquire about prognosis at the points of diagnosis, staging, post-treatment, and end-of-life (ORs ≥ 1.41). Caregivers’ inquiries that referenced digestive/gastrointestinal, genitourinary, head and neck, hematologic, lung, or musculoskeletal cancers had greater odds of a prognosis inquiry (ORs ≥ 1.50). Caregivers’ inquiries that discussed chemotherapy were associated with greater odds of a prognosis inquiry as compared with inquiries that did not discuss chemotherapy (OR = 1.85, 95% CI [1.29, 2.67]). The odds of a prognosis inquiry were also higher when concurrent discussion topics focused on general questions about cancer (OR = 1.35, 95% CI [1.12, 1.64]) or cancer treatment (OR = 1.96, 95% CI [1.65, 2.32]). The odds of a prognosis inquiry were lower when concurrent discussion topics focused on finding healthcare services (OR = 0.31, 95% CI [0.24, 0.40]).

**Table 4:** Logistic regressions of factors associated with prognosis inquiries to the CIS among caregivers (*n* = 44,344)

	OR	95% CI	
Language			
Spanish	2.05*	1.69	2.47
English (ref.)	-	-	-
Communication channel			
LiveHelp	1.44*	1.22	1.71
Email	0.72*	0.55	0.95
Social media	0.73	0.39	1.36
Telephone (ref.)	-	-	-
Cancer continuum phase			
Diagnosis	1.41*	1.04	1.91
Staging	3.28*	2.46	4.37
Post-treatment	1.58*	1.14	2.18
End-of-life	2.24*	1.70	2.97
Treatment (ref.)	-	-	-
Cancer sites^1^			
Breast	1.02	0.74	1.41
Digestive/gastrointestinal	1.50*	1.18	1.90
Genitourinary	1.77*	1.34	2.34
Gynecologic	1.37	0.98	1.91
Head and neck	1.69*	1.12	2.54
Hematologic	1.62*	1.21	2.16
Lung	1.52*	1.13	2.05
Musculoskeletal	1.75*	1.18	2.60
Neurologic	1.18	0.80	1.74
Discussion topics^1^			
Chemotherapy	1.85*	1.29	2.67
Coping	1.29	0.91	1.81
Finding healthcare services	0.31*	0.24	0.40
General cancer questions	1.35*	1.12	1.64
General treatment questions	1.96*	1.65	2.32

*Statistically significant values (*α* ≤ 0.05)

^1^For each cancer site and discussion topic, the reference category is the absence of that specific cancer site or discussion topic within a CIS inquiry. For example, the reference category for breast cancer is a CIS inquiry that did not mention breast cancer

## Discussion

Using contact data from a national cancer information service, this study identified and described cancer prognostic information-seeking among two key groups directly impacted by cancer: survivors and caregivers. Although prognosis inquiries represent a small proportion of all CIS inquiries, the present findings suggest that survivors and caregivers seek prognosis information from additional resources beyond healthcare providers. Importantly, the CIS encourages follow-up discussions with the client’s healthcare team about prognosis, as seen in the number of referrals to healthcare providers when prognosis information is shared during CIS interactions. In doing so, the CIS supports survivors’ and caregivers’ dialogue with healthcare professionals regarding how the information shared by the CIS may or may not apply to the individual’s specific situation [32]. In addition, the CIS refers to other national support organizations that may assist with the psychosocial needs of individuals seeking prognosis information, including the Cancer Support Community, Cancer Hope Network, and CancerCare.

Results from descriptive analyses and logistic regressions complement—and add to—previous research highlighting the varied information needs of survivors and caregivers with respect to cancer prognosis [3, 17–21]. Notably, these analyses show that almost two-thirds of prognosis inquiries to the CIS are made by caregivers who may be seeking information for themselves and/or their loved ones. That caregivers sought prognosis information from the CIS more commonly than survivors emphasizes the importance of ensuring reputable information resources about prognosis continue to be available to caregivers outside of clinical settings to aid in understanding their loved ones’ cancer prognosis.

Results further indicate that both survivors and caregivers frequently inquired about prognosis via the CIS’ telephone and LiveHelp (web-based instant messaging) channels, suggesting their potential preference for the expediency of information receipt and that the CIS’ provision of real-time information is a useful feature for complementing survivors’ and caregivers’ clinical communication encounters with their oncology care teams. Moreover, the emergence of LiveHelp as a frequently used channel supports previous research highlighting the increased use of the internet to meet individuals’ demands for quick and easily accessible cancer information [3].

Logistic regression analyses identified several factors associated with prognosis inquiries for both survivors and caregivers. The finding that CIS inquiries occurring in Spanish were associated with prognosis discussions suggests caregivers and survivors who speak Spanish as a primary language may face heightened challenges to obtaining needed cancer prognosis information, perhaps intensified by barriers to language-concordant cancer information in clinical settings [37]. The study also identified contexts in which caregivers and survivors inquired about cancer prognosis—specifically, within discussions about chemotherapy and general cancer treatment, possibly indicating individuals may have questions about treatment effectiveness and, ultimately, cancer survival outcomes. Discussions focused on finding healthcare services were negatively associated with prognostic inquiries for both groups, suggesting that if the uncertainties motivating people to contact the CIS are more practical in nature (i.e., accessing healthcare), they are less likely to focus on prognosis.

Findings also revealed survivors’ and caregivers’ unique prognosis information-seeking profiles. For example, the proportion of survivors’ prognosis inquiries that included discussion about the post-treatment phase of cancer care (19.1%) was relatively high when considered alongside the proportion of post-treatment references in caregivers’ prognosis inquiries (5.8%). This may serve as an indication of survivors wanting to know if their cancer is cured, long-term survival rates following treatment completion, and/or likelihood of disease recurrence. Conversely, the proportion of caregivers’ prognosis inquiries about the end-of-life cancer care phase (8.5%) was higher than the proportion of survivors’ inquiries about end-of-life (1.8%), reflecting previous findings that caregivers’ information needs may increase as the illness progresses. Overall, study findings reinforce the value of exchanging prognosis-related communication among triads of providers, caregivers, and survivors across key phases of the cancer continuum [8]. Inquiries focused on general cancer questions were also associated with prognosis inquiries for caregivers, indicating a need to learn more about their loved one’s disease. Related to specific cancer types, findings for each group were variable, likely due to myriad factors such as stage of disease (or recurrence), treatment regimens, and caregiving burden.

### Limitations, strengths, and future directions

These findings should be considered within the context of the study’s limitations and strengths. First, the CIS dataset represents a convenience sample of individuals who proactively choose to make health-related information inquiries to a national cancer information service. This may limit the generalizability of the findings to broader populations. For example, there is variation among cancer survivors and caregivers in whether—and what type of—prognosis information is desired [11, 38] as well as their preferences for mode of receiving cancer information (e.g., interest in seeking cancer information via LiveChat or the internet) [27, 28]. The current sample likely does not reflect the totality of prognosis information preferences of survivors and caregivers. Also, as CIS data are coded and aggregated, qualitative investigation of prognosis inquiries to identify what is specifically discussed is not feasible. Second, due to federal Office of Management and Budget limitations on the type and proportion of CIS users that can be queried for sociodemographic information, the current study did not allow for an investigation of sociodemographic variations associated with prognosis inquiries. Third, the cross-sectional data limit causal inferences about the associations found in this study. Study limitations are countered by notable strengths, including the large number of CIS inquiries coded over a multi-year period, adding reliability to inferences made from aggregated analyses. Additionally, the nature of the CIS as a professional resource responding to information requests in real time makes the data valuable in terms of ecological validity in that interactions represent a real-world laboratory of health communication and cancer information-seeking [38].

Future directions include continued investigation into the cancer prognosis information needs of caregivers and survivors, specifically through mixed-methods and qualitative research, to better describe these needs and help providers and public-facing services more effectively address them. In addition, examination of survivors’ and caregivers’ longitudinal patterns of prognosis information-seeking across time and the cancer continuum is warranted. Such longitudinal research can also yield insights into the sources of information utilized over time [31]. These sources include healthcare professionals, family and friends, and national organizations as well as new and emerging sources such as artificial intelligence tools and platforms [39, 40].

### Conclusion

Findings from this CIS study raise the collective understanding of the information needs of cancer survivors and caregivers pertaining to cancer prognosis and offer suggestions on how best to meet them, including delivery of language-concordant cancer information across multiple communication channels, provision of prognosis information throughout the trajectory of cancer care [12–14], and promotion of patient-caregiver-provider communication. Publicly available services, such as the CIS, can serve as trusted [26], reliable, and evidence-based cancer information resources to survivors and caregivers, complementing discussions with providers [41]. Themes in how cancer survivors and caregivers seek prognosis information highlight areas that can be used to guide information provision and dissemination of prognosis-related messaging to these key groups. By helping to meet cancer prognosis information needs, the CIS and other public information resources may ultimately help improve survivors’ and caregivers’ psychosocial well-being [1–6].

## Data Availability

Study data may be made available from the authors upon reasonable request and with the permission of NCI’s Cancer Information Service.

## Declarations

### Disclaimer

The opinions expressed by the authors are their own, and this material should not be interpreted as representing the official viewpoint of the U.S. Department of Health and Human Services, the National Institutes of Health, or the National Cancer Institute.
